# Psychotherapeutic Treatments of Trichotillomania

**DOI:** 10.1192/j.eurpsy.2022.1655

**Published:** 2022-09-01

**Authors:** N. Mhaimeed, P. Sinha

**Affiliations:** 1 Weill Cornell Medicine Qatar, Medical Education, Education City, Qatar; 2 Weill Cornell Medicine - Qatar, Qatar Foundation - Education City, Doha, Qatar

**Keywords:** obsessive-compulsive disorder, Trichotillomania

## Abstract

**Introduction:**

Trichotillomania (TTM), also known as hair pulling disorder, is an obsessive- compulsive disorder characterized by the recurrent, overwhelming urge to repeatedly pull out one’s hair. Hair pulling can occur anywhere on the body but is most common on the scalp, eyebrows, and eyelashes and subsequently results in bald patches. While TTM is a very prevalent, debilitating disorder, there is still no FDA approved treatment that exists.

**Objectives:**

The main objective of this study is to explore the various forms of available psychotherapy available for the treatment of trichotillomania.

**Methods:**

Two independent reviewers conducted title, abstract, full-text searching and data extraction among the PubMed, PsycINFO, and ResearchGate data bases. Of the 79 articles screened, five were included in this review

**Results:**

Habit reversal therapy (HRT) is a form of cognitive behavioral therapy that is considered the first line treatment for management of TTM. Other psychotherapeutic techniques include acceptance and commitment therapy, progressive muscle relaxation, and mindfulness therapy.

**Conclusions:**

This study supports the current data which states that HRT is the first line treatment and there is yet to be a pharmacological treatment of choice for TTM. It is also very important to note that TTM is still underdiagnosed and can be mistaken for a dermatological disorder like alopecia aerata. Furthermore, many people with trichilemmoma have underlying mental health disorders such as anxiety and depression that must first be addressed before treating the hair pulling itself.

**Disclosure:**

No significant relationships.

